# Is there a danger for myopia in anti-doping education? Comparative analysis of substance use and misuse in Olympic racket sports calls for a broader approach

**DOI:** 10.1186/1747-597X-6-27

**Published:** 2011-10-11

**Authors:** Miran Kondric, Damir Sekulic, Andrea Petroczi, Ljerka Ostojic, Jelena Rodek, Zdenko Ostojic

**Affiliations:** 1 Faculty of Sport; University of Ljubljana, Gortanova 11, Ljubljana - 10000, Slovenia; 2 Faculty of Kinesiology; University of Split, Teslina 6, Split - 21000, Croatia; 3 NIHON doo, Spinutska 65, Split - 21000, Croatia; 4 School of Life Sciences, Kingston University London, Penrhyn Road, KT1 2EE, UK; 5 School of Medicine; University of Mostar, Bijeli brijeg, Mostar - 63000, Bosnia and Herzegovina

**Keywords:** racket sport, anti-doping, drug, athlete, drinking, supplements

## Abstract

**Background:**

Racket sports are typically not associated with doping. Despite the common characteristics of being non-contact and mostly individual, racket sports differ in their physiological demands, which might be reflected in substance use and misuse (SUM). The aim of this study was to investigate SUM among Slovenian Olympic racket sport players in the context of educational, sociodemographic and sport-specific factors.

**Methods:**

Elite athletes (N = 187; mean age = 22 ± 2.3; 64% male) representing one of the three racket sports, table tennis, badminton, and tennis, completed a paper-and-pencil questionnaire on substance use habits. Athletes in this sample had participated in at least one of the two most recent competitions at the highest national level and had no significant difference in competitive achievement or status within their sport.

**Results:**

A significant proportion of athletes (46% for both sexes) reported using nutritional supplements. Between 10% and 24% of the studied males would use doping if the practice would help them achieve better results in competition and if it had no negative health consequences; a further 5% to 10% indicated potential doping behaviour regardless of potential health hazards. Females were generally less oriented toward SUM than their male counterparts with no significant differences between sports, except for badminton players. Substances that have no direct effect on sport performance (if timed carefully to avoid detrimental effects) are more commonly consumed (20% binge drink at least once a week and 18% report using opioids), whereas athletes avoid substances that can impair and threaten athletic achievement by decreasing physical capacities (e.g. cigarettes), violating anti-doping codes or potentially transgressing substance control laws (e.g. opiates and cannabinoids). Regarding doping issues, athletes' trust in their coaches and physicians is low.

**Conclusion:**

SUM in sports spreads beyond doping-prone sports and drugs that enhance athletic performance. Current anti-doping education, focusing exclusively on rules and fair play, creates an increasingly widening gap between sports and the athletes' lives outside of sports. To avoid myopia, anti-doping programmes should adopt a holistic approach to prevent substance use in sports for the sake of the athletes' health as much as for the integrity of sports.

## Background

The fact that athletes routinely use a wide array of substances is well documented [1]as are the potential reasons for use [2]. Whilst performance-enhancing substances are recognised in global as well as local anti-doping prevention programmes [3], other substances such as alcohol, tobacco and psychoactive drugs constitute a somewhat neglected area in the current idealised anti-doping educational effort. This prevailing approach creates an artificial divide between athletes' lives as sportspersons and private individuals. In reality, athletes constantly navigate their athletically active years on a tightrope between the different expectations they face both as athletes (often being in the spotlight) and as ordinary citizens, and know that failing in one part of their lives could easily result in failures in the other and vice versa.

Substances in sports are mainly used for the following reasons: (1) enhancing physical capacities (e.g., enhancing endurance, strength, or recovery between exercise sessions); (2) psycho-stimulation (e.g., as a way of dealing with psychological stress); or (3) improving physical appearance (e.g., for achieving a lean figure) [4-6]. Contemporary sport legislation recognises two types of substances used in sports: (1) non-controlled substances, such as the majority of nutritional supplements, and (2) products that contain prohibited substances (the use of which is often referred to as 'doping'). Nutritional supplementation is defined as a preparation intended to provide nutrients, such as vitamins, minerals, fibre, fatty acids or amino acids, which are otherwise missing or not consumed in a sufficient quantity in the athlete's diet [7]. Doping is defined as the occurrence of one or more anti-doping code violations, mostly detected by the presence of a prohibited substance or its metabolites or markers in an athlete's specimens [8]. Whereas nutritional supplementation should be considered a logical and natural consequence of the increased physical demands on athletes [9,10], doping is deemed unethical for performance enhancement [11]. Excessive use of NS and polypharmacy [12] as well as doping hasbeen connected to serious health problems [13,14] and even death [15].

It is generally accepted that substance use and misuse (SUM) in sports is more common in physically demanding sports (e.g., weightlifting or cycling) than in sports that require advanced specific motor skills (e.g., diving, sailing, table tennis or curling) [4,16]. However, to our knowledge, such generalisation is not sufficiently supported by any systematic comparative analyses of SUM across a variety of sports. Instead, the association of doping with particular types of sports has mostly come from anti-doping testing and the consequential public perception about doping in certain sports such as professional cycling, track and field or weightlifting.

In its capacity as the global anti-doping governing body, the World Anti-Doping Agency (WADA) established a detection-based system consisting of both systemic and random testing of athletes' blood and/or urine. Participation in this system is mandatory for all athletes registered in the testing pool at the national Olympic committees. This component of WADA's anti-doping programme uses *detection *and *sanctions *to keep doping out of sports by random testing from *a pool of selected athletes *in and out of the competition to find evidence for the presence of a prohibited substance or substances. Those with confirmed positive test results are typically banned from competition for a period of time (1-2 years) and stripped of any medals and records that were thought to have been achieved with the aid of doping. As a different approach, WADA's anti-doping prevention aims to create a strong anti-doping culture and target *all athletes *with its *value-based *education programmes to foster abstinence from prohibited performance-enhancing drugs.

The majority of sport activities take place outside of controlled environments, leading to substance use without medical advice or supervision [17]. The mismatch in targets in the anti-doping prevention and deterrence programmes coupled with the limited concern over substances such as alcohol and social drugs raises questions about the suitability of the current anti-doping policy.

Whilst both arms of WADA's anti-doping effort represent heroic measures to keep doping out of sports, laboratory statistics shows no significant change between 2003 and 2009 with the proportion of adverse and atypical findings ranging between 1.50% and 2.12% [15]. Self-reports, alternative analyses and epidemilogic estimations indicate that the actual prevalence of doping is greater than this official statistic and ranges up to 40% [18]. Although it is difficult to make a direct comparison between the latter and the WADA laboratory report, a recently published report evaluating 7,289 blood samples from 2,737 track and field athletes in the athlete testing pool, using the Athlete Biological Passport approach [19], estimated the prevalence of blood doping to be at 14% overall and between 1% and 48% for sub-populations [20], which supports the results from the epidemiologic studies.

Furthermore, it is apparent from annual lab statistics [8] that the doping-testing programmes concentrate the analyses on(a) Olympic rather than in Non-Olympic sports, and on(b) sports which are already associated with doping (e.g., 'physically demanding sports'). For example, in 2009, 26,593 urine and blood samples from track and field athletes yielded 398 total findings of suspicious substances. At the same time, 467 tests of curling athletes resulted in only 14 total findings [21]. The higher absolute number of adverse or atypical analytical findings in 'highly physically demanding' track and field athletics than in 'less-demanding' curling seems unsurprising (398 vs.14). But, the surprising pattern is evident for aquatics (13,995 total samples; 156 total findings, or 0.65% of samples) in comparison to shooting (24/2,630; 0.91% of samples) or archery (14/976; 1.44% of samples). Doping in Olympic racket sports was found to range from0.17% to 0.94% in the following order: badminton: 2/1,175, tennis: 17/3,945 and table tennis: 10/1,066.

Racket sport games are characterised by a handheld racket that is used to propel a missile between two (or four) players with the purpose of placing the missile in such a position that one player is unable to return it successfully. These sports are also characterised by an area of play that has a specified size, within which the missile must be contained, and by the presence of a net that the missile must pass above on each play. The unique sizes and shapes of the area of play, the height of the net and the type of missile and racket used give character to each variant of the game. Racket sports are unique due to the fact that players can modify the physiological demands of the game by controlling the rest intervals between rallies, games and sets. Average oxygen consumption for single-match duration badminton is reported to be 39.6 ± 5.7 ml/kg/min (73% VO2max); oxygen consumption for table tennis is 26 ± 4 ml/kg/min (47% of VO2 max), and consumption for tennis is 29 ± 6 ml/kg/min (51% of VO2 max). Of course, average match duration must also be considered. In the 2006 badminton World Championship in Madrid, the average match duration was 33:35 minutes. At the Olympic Games in Beijing, the average table tennis match lasted for 27:31 minutes. The average duration of tennis matches depends on the type of court but typically ranges from 120 to 180 minutes. For example, at Wimbledon in 2005, the average duration of tennis matches was 137 minutes, whereas the average match in the Australian Open that same year lasted for 154 minutes. A major determinant of the outcome of a game is an individual's physical fitness, which can be influenced by SUM [22,23].

Although SUM is regularly investigated in sports as a whole [24], SUM is rarely studied in racket sports. To the best of our knowledge, apart from studies dealing with sports and physical activity in youth and related SUM issues in which racket sports (tennis mostly) were not studied systematically [25,26], there are only a few papers addressing SUM in Olympic racket sports. Briefly, Kondric et al. [27] reported on SUM habits in Slovenian table tennis players. Also, Maquirriain [25] analysed offences to the Doping Code committed by tennis players between 2003 and 2009. When studying SUM problems in sports, previous investigations noted that SUM is gender-, sociodemographic-, culture-, and sport-specific and, therefore should be studied accordingly [24]. Furthermore, one recently published study highlighted the advantage of a comparative approach to investigating SUM in sports [28]. The authors investigated SUM templates in aesthetic sports (synchronised swimming, dance sport, and ballet) while focusing on organisational differences and anti-doping regulations across the different activities. In short, the authors found evidence for (1) different substance use and misuse patterns and (2) sport-specific correlations between educational and sociodemographic factors along with the likelihood of overall substance misuse (as predictors) and doping. These findings demonstrated the necessity of using a similar experimental approach when evaluating other sports and sport activities.

Apart from the fact that SUM is rarely investigated among racket sports, we determined that these sports would be particularly suitable for our study based on several factors. First, table tennis, tennis and badminton are Olympic Sports that fall directly under WADA jurisdiction and anti-doping legislation. Second, all three sports share similar competitive characteristics as they are all individual sports with no physical contact between opponents. However, these sports are also diverse in terms of physiological demands.

The primary aim of this study was to investigate substance use and misuse and to determine sport-specific and sex-related differences regarding SUM habits and attitudes in Slovenian Olympic racket sports (table tennis, badminton and tennis). In addition, we have studied the relationships between sports, education, and non-sport-specific substance use and misuse with doping factors. In response to the recognised need for critical evaluation of the current anti-doping approach and to extend the harm reduction argument presented in the literature for doping control [29] to include substances beyond performance-enhancing drugs, the present study evaluated non-sport-specific substance use and misuse among elite racket sport players in order to ascertain whether the current anti-doping policy has the potential to adequately address its mission of protecting the integrity of sport as well as the health of athletes.

## Materials and methods

We studied a total of 188 participants divided into three groups: table tennis players (N = 78), badminton players (N = 83), and tennis players (N = 27). All players were 18 years of age or older and had participated in at least one of the two most recent competitions at the highest national level for their sport (e.g., Slovenian Nationals). The number of T is almost half the size of the other two groups because mature tennis players (+18) are typically professionals and rarely compete in the Slovenian Nationals. Participants were informed of the purpose and aim of this study and participated voluntarily. Participants were informed that they could refuse to participate and could withdraw from the study at any time without giving a reason or explanation. Participation was strictly anonymous with no personal data collected regarding date of birth, city of residence, or occupation. Answer options were presented as multiple-choice closed responses for all of the questions. SUM and its corresponding educational, sociodemographic, and sport-specific factors were investigated using a previously developed and validated questionnaire for studying SUM [27,28,30]. The authors are available for any further information about the details and the measuring tools used in this investigation. The sociodemographic data collected included age, sex, and educational level, whereas sport factors included sport experience (in age), sport status (amateur, semiprofessional, or professional), and sport achievement (based on a 6-point scale using results from local competitions to international achievements). Substance use and misuse data consisted of questions on binge drinking (7-point scale from "I do not drink alcohol" to "I binge a few times a week"), cigarette smoking (7-point scale from "not smoking" to "2+packs daily"), consumption of drugs and oppiates (use of different drugs and opiates were inquired after but subjects reported only marihuana and hashish use). Doping factors were evaluated with questions concerning the athlete's opinions on doping practice in their sport (4-point scale from "I do not think doping is used" to "Doping is often"), potential doping habits (4-point scale from "I do not intend to use doping" to "I'll use it if assured it will help me"), and trust in their coach regarding doping and trust in their physician regarding doping (both nominal "yes-no" questions). Nutritional supplements were reported separately and included the consumption of isotonics, proteins, carbohydrates, and recovery supplements. Additionally, we asked athletes to indicate who advised them to use NS with the coach, physician, friend, and self-decided as choices. The key questions and answer options are shown in Tables.

Counts (frequencies) and proportions were calculated for all data. Owing to the measurement levels present in the data, nonparametric Kruskal-Wallis ANOVA test was applied to establish differences between the sports for each of the observed variables. Spearman's rank-order correlation was calculated between ordinal predictors and SUM criteria. The statistical significance level of 95% (p < 0.05) was applied. Statistical analyses were performed using Statsoft's STATISTICA version 7.

## Results

The female racket sport athletes were the same age and had the same educational level. Although there are differences in duration of sport engagement (time an athlete has played the sport), with badminton players being involved in their sport for a shorter period of time, there was no difference in competitive achievement (defined as results from competitions) and status (e.g., amateur, semipro, or professional level) between the three sports: between one-third and one-half of the players were semi-professionals (Table 1). Badminton players reported the highest level of binge drinking. Statistically significant differences were found in cigarette use, but this was mostly because of the high proportion of smoking cessation among badminton and tennis players (Table 2). No significant differences were found for doping factors, although 1 in 10 badminton players said they would use doping if they were assured that it will improve their performance without any negative health consequences. Data revealed that most female athletes do not trust their own coaches regarding doping issues (mistrust in coaches ranges from 61% in badminton to 83% in tennis), whereas their trust in their physicians' opinions on the same issue was somewhat higher (Table 3). Approximately 50% of females declared no use of nutritional supplementation. Female racket sport athletes reported using vitamins, minerals and isotonics almost exclusively. The reported use of other substances was very low among tennis players who were mostly advised by coaches or medical professional to consume nutritional supplements, whereas athletes in table tennis and badminton were not (Table 4).

**Table 1:** Sociodemographic and sport factors and differences between racket sports

	MALES				FEMALES			
	
	T	TT	B	K-W (p)	T	TT	B	K-W (p)
	f(%)	f(%)	f(%)		f(%)	f(%)	f(%)	
**Age (years)**				0.73 (0.69)				1.15 (0.46)
19-22	7(33.3)	23(46.0)	22(43.1)		4(66.7)	13(46.4)	18(58.1)	
22-25	8(38.1)	10(20.0)	6(11.8)		2(33.3)	6(21.4)	5(16.1)	
25-28	2(9.5)	5(10.0)	5(9.8)		0(0.0)	5(17.9)	2(6.5)	
28+	4(19.0)	12(24.0)	18(35.3)		0(0.0)	4(14.3)	6(19.4)	
**Education (school)**				10 (0.01)				3.4 (0.18)
Elementary	3(14.3)	13(26.0)	4(7.8)		2(33.3)	2(7.1)	8(25.8)	
High	12(57.1)	17(34.0)	17(33.3)		1(16.7)	9(32.1)	10(32.3)	
Student	4(19.0)	13(26.0)	11(21.6)		2(33.3)	9(32.1)	9(29.0)	
Graduated	2(9.5)	7(14.0)	19(37.3)		1(16.7)	7(25.0)	4(12.9)	
**Sport experience**				3 (0.20)				8.4 (0.02)
less than 5 years	0(0.0)	1(2.0)	1(2.0)					
5 to 10 years	8(38.1)	7(14.0)	17(33.3)		2(33.3)	4(14.3)	14(45.2)	
10 to 15 years	7(33.3)	20(40.0)	12(23.5)		2(33.3)	13(46.4)	13(41.9)	
15 +	6(28.6)	22(44.0)	21(41.2)		2(33.3)	11(39.3)	4(12.9)	
**Sport status**				16 (0.01)				1.56 (0.45)
Amateur	11(52.4)	14(28.0)	35(68.6)		3(50.0)	14(50.0)	20(64.5)	
Semiprofessional	9(42.9)	28(56.0)	13(25.5)		3(50.0)	13(46.4)	11(35.5)	
Professional	1(4.8)	8(16.0)	3(5.9)		0(0.0)	1(3.6)	0(0.0)	
**Sport achievement**				0.13 (0.93)				1.79 (0.40)
local competition	1(4.8)	0(0.0)	4(7.8)		0(0.0)	0(0.0)	1(3.2)	
local achievement	2(9.5)	0(0.0)	5(9.8)		0(0.0)	0(0.0)	3(9.7)	
national competition	2(9.5)	5(10.0)	4(7.8)		2(33.3)	4(14.3)	7(22.6)	
national achievement	2(9.5)	12(24.0)	6(11.8)		1(16.7)	4(14.3)	5(16.1)	
international competition	6(28.6)	22(44.0)	14(27.5)		1(16.7)	14(50.0)	7(22.6)	
international achievement	8(38.1)	11(22.0)	18(35.3)		2(33.3)	6(21.4)	8(25.8)	

T - tennis; TT - table tennis; B - badminton; frequencies - f, percentage - %; KW - Kruskall-Wallis test; p - statistical significance for df = 2

**Table 2:** Substance use and misuse data and differences between racket sports

	MALES				FEMALES			
	
	T	TT	B	K-W (p)	T	TT	B	K-W (p)
	f(%)	f(%)	f(%)		f(%)	f(%)	f(%)	
**Binge drinking**				24.01 (0.01)				10 (0.01)
I do not drink alcohol	1(4.8)	3(6.0)	1(2.0)		4(66.7)	6(21.4)	4(12.9)	
Never. although I consume alcohol	1(4.8)	21(42.0)	2(3.9)		0(0.0)	6(21.4)	1(3.2)	
rarely	7(33.3)	6(12.0)	8(15.7)		1(16.7)	7(25.0)	7(22.6)	
a few times a year	4(19.0)	10(20.0)	10(19.6)		0(0.0)	6(21.4)	4(12.9)	
once a month or so	2(9.5)	8(16.0)	18(35.3)		0(0.0)	3(10.7)	10(32.3)	
once a week	4(19.0)	2(4.0)	11(21.6)		0(0.0)	0(0.0)	5(16.1)	
a few times a week	2(9.5)	0(0.0)	1(2.0)		1(16.7)	0(0.0)	0(0.0)	
**Cigarette smoking**				24.05 (0.01)				29.9 (0.01)
Not at all	0(0.0)	34(68.0)	1(2.0)		0(0.0)	22(78.6)	0(0.0)	
I quit	13(61.9)	3(6.0)	42(82.4)		6(100.0)	3(10.7)	25(80.6)	
from time to time	4(19.0)	4(8.0)	5(9.8)		0(0.0)	3(10.7)	5(16.1)	
less than 10 cigs per day	1(4.8)	5(10.0)	1(2.0)		(0.0)	(0.0)	(0.0)	
10-20 cigs per day	1(4.8)	2(4.0)	2(3.9)		0(0.0)	0(0.0)	1(3.2)	
1-2 packs per day	1(4.8)	0(0.0)	0(0.0)					
2+ packs per day	1(4.8)	2(4.0)	0(0.0)					
**Cannabinoids and opiates**				2.7 (0.26)				2.5 (0.28)
No	16(76.0)	45(90.0)	41(41.0)		6(100.0)	20(71.0)	25(25.0)	
Yes, occasionally (marijuana and hashish)	5(23.0)	5(10.0)	10(10.0)		0(0.0)	8(28.0)	6(6.0)	

T - tennis; TT - table tennis; B - badminton; frequencies - f, percentage - %; KW - Kruskall-Wallis test; p - statistical significance for df = 2

**Table 3:** Doping factors and differences between sports

	MALES				FEMALES			
	
	T	TT	B	K-W (p)	T	TT	B	K-W (p)
	f(%)	f(%)	f(%)		f(%)	f(%)	f(%)	
**Doping in sport**				3.3 (0.19)				4.2 (0.12)
I don't think that it is used	5(23.8)	11(22.0)	5(9.8)		1(16.7)	8(28.6)	2(6.5)	
Don't know - not familiar	10(47.6)	20(40.0)	22(43.1)		3(50.0)	8(28.6)	10(32.3)	
It is used but rarely	2(9.5)	15(30.0)	19(37.3)		2(33.3)	11(39.3)	17(54.8)	
Doping is often	3(14.3)	4(8.0)	5(9.8)		0(0.0)	1(3.6)	2(6.5)	
**Potential doping habits**				1.5 (0.47)				3.2 (0.20)
I do not intend to use doping	11(52.4)	33(66.0)	29(56.9)		6(100.0)	21(75.0)	20(64.5)	
Not sure about it	3(14.3)	7(14.0)	13(25.5)		0(0.0)	5(17.9)	8(25.8)	
I will use it if it will help me with no health hazard	5(23.8)	7(14.0)	6(11.8)		0(0.0)	1(3.6)	3(9.7)	
If assured it will help me no matter to health hazard	2(9.5)	3(6.0)	3(5.9)		0(0.0)	1(3.6)	0(0.0)	
Trust in coach regarding doping*								
No, I don't trust him/her	13(61.9)	44(88.0)	34(66.7)		5(83.3)	21(75.0)	19(61.3)	
Yes	8(38.1)	6(12.0)	17(33.3)		1(16.7)	7(25.0)	12(38.7)	
Trust in physician regarding doping*								
No, I don't trust him/her	17(81.0)	40(80.0)	31(60.8)		3(50.0)	21(75.0)	18(58.1)	
Yes	4(19.0)	10(20.0)	20(39.2)		3(50.0)	7(25.0)	13(41.9)	

T - tennis; TT - table tennis; B - badminton; frequencies - f, percentage - %; KW - Kruskall-Wallis test; p - statistical significance for df = 2; * denotes nominal variables where differences between sports were not calculated

**Table 4:** Nutritional supplementation (NS) data (T - tennis; TT - table tennis; B - badminton; frequencies - f, percentage - %)

	MALES			FEMALES		
	
	T	TT	B	T	TT	B
	f(%)	f(%)	f(%)	f(%)	f(%)	f(%)
**Isotonics and vitamins**						
No	8(38.1)	37(74.0)	32(62.7)	3(50.0)	18(64.3)	16(51.6)
Yes	13(61.9)	13(26.0)	19(37.3)	3(50.0)	10(35.7)	15(48.4)
**Proteins**						
No	12(57.1)	45(90.0)	37(72.5)	6(100.0)	26(92.9)	28(90.3)
Yes	9(42.9)	5(10.0)	14(27.5)	0(0.0)	2(7.1)	3(9.7)
**Carbohydrates**						
No	11(52.4)	46(92.0)	45(88.2)	6(100.0)	28(100.0)	31(100.0)
Yes	10(47.6)	4(8.0)	6(11.8)			
**Recovery supplements**						
No	21(100.0)	49(98.0)	45(88.2)	6(100.0)	28(100.0)	31(100.0)
Yes	0(0.0)	1(2.0)	6(11.8)			
**NS in general**						
Yes	15(71.4)	14(28.0)	27(52.9)	3(50.0)	11(39.3)	16(51.6)
No	6(28.6)	36(72.0)	24(47.1)	3(50.0)	17(60.7)	15(48.4)
**Advised by coach to use NS**						
No	13(46.7)	49(92.9)	40(59.3)	5(66.7)	28(100.0)	29(87.5)
Yes	8(53.3)	1(7.1)	11(40.7)	1(33.3)	0(0.0)	2(12.5)
**Advised by physician to use NS**						
No	21(100.0)	50(100.0)	46(81.5)	4(33.3)	26(81.8)	29(87.5)
Yes	0(0.0)	0(0.0)	5(18.5)	2(66.7)	2(18.2)	2(12.5)
**Advised by friend to use NS**						
No	17(73.3)	47(78.6)	43(70.4)	6(100.0)	23(54.5)	27(75.0)
Yes	4(26.7)	3(21.4)	8(29.6)	0(0.0)	5(45.5)	4(25.0)
**Self decided to use the NS**						
No	16(66.7)	38(14.3)	40(59.3)	6(100.0)	23(54.5)	22(43.70)
Yes	5(33.3)	12(85.7)	11(40.7)	0(0.0)	5(45.5)	9(56.3)

T - tennis; TT - table tennis; B - badminton; frequencies - f, percentage - %

Table tennis players in this sample were the most advanced in their careers, although we found no significant difference in other sport-related factors, such as the results achieved (Table 1). Similar to their female counterparts, male badminton players binge drink more than thosein tennis and much more than table tennis players. Significant differences in cigarette smoking showed the highest incidence among tennis players (Table 2). In male athletes, there was no statistically significant difference between players of different racket sports in their perception of doping behaviours. One-third of the studied athletes thought that doping is used in their sport. 60% to 90% of the male athletes reported that they do not trust coaches' or medics' opinions regarding doping issues and problems. A minority of athletes (10%in badminton, 15% in table tennis and 24% in tennis) indicated that they would use doping if assured that it would help them achieve competitive results without any negative health consequences. However, 5% to 10% of the studied male athletes declared that they might potentially dope regardless of the possible health hazard (Table 3). Nutritional supplement use was mostly frequently reported by tennis players, followed by badminton and table tennis. More than half of the tennis and badminton players were formally advised by a coach or medical professional to use nutritional supplements (Table 4).

Players in this sample reported varied levels of substance use with binge drinking and cannabinoids use reaching a concerning level with 40% and 30% binge drinking and 16% and 21% using cannabinoids, form males and females respectively. This level of use is comparable with the national statistics as detailed below. Tobacco use, in contrast, appears to be a male phenomenon. Owing to the dissimilarities between males and females regarding their SUM, the correlations between the studied ordinal variables were calculated separately for males (Table 5) and females (Table 6).

**Table 5:** sSport (T - tennis; TT - table tennis; B - badminton) specific correlation analysis between ordinal variables for males

		Education	Binging	Smoking	Sport experience	Sport status	Sport achievement	Doping in sport
Age	T	0.53*						
	TT	0.41*						
	B	0.63*						

Binging	T	0.09						
	TT	-0.11						
	B	-0.49*						

Smoking	T	-0.01	0.34					
	TT	-0.21	0.59*					
	B	-0.32*	0.34*					

Sport experience	T	0.36	-0.28	-0.14				
	TT	0.42*	0.05	0.16				
	B	0.65*	-0.26	-0.08				

Sport status	T	0.07	0.00	-0.0	0.06			
	TT	0.04	0.01	0.03	0.43*			
	B	0.20	-0.07	-0.02	0.31*			

Sport achievement	T	0.04	-0.07	-0.24	0.26	0.46*		
	TT	-0.08	0.08	-0.02	0.14	0.29*		
	B	0.06	-0.14	-0.2	0.13	0.40*		

Doping in sport	T	-0.38	0.24	0.1	-0.02	-0.33	-0.33	
	TT	-0.01	0.00	0.06	-0.21	-0.1	-0.18	
	B	-0.15	0.05	0.26	-0.080.44*	-0.38*	-0.25	

Potential doping habits	T	-0.15	0.15	0.29	-0.24	0.20	-0.03	-0.38
	TT	-0.14	0.08	0.13	-0.13	0.32*	-0.09	0.39*
	B	-0.16	0.22	0.12	-0.14	0.23	-0.06	-0.18

* denotes significant correlation coefficients at p < 0.05

**Table 6:** Sport (T - tennis; TT - table tennis; B - badminton) specific correlation analysis between ordinal variables for females

		Education	Binging	Smoking	Sport experience	Sport status	Sport achievement	Doping in sport
Age	T	0.75*						
	TT	0.59*						
	B	0.76*						

Binging	T	0.78*						
	TT	-0.43*						
	B	-0.01						

Smoking	T	#	#					
	TT	-0.10	0.19					
	B	0.09	0.31					

Sport experience	T	0.92*	0.49	#				
	TT	0.52*	-0.24	-0.12				
	B	0.10	0.02	0.07				

Sport status	T	-0.90*	-0.69	#	-0.82*			
	TT	-0.01	0.07	0.15	0.17			
	B	-0.49*	-0.22	-0.16	0.30			

Sport achievement	T	0.23	0.57	#	0.00	-0.10		
	TT	-0.21	0.07	0.17	0.18	0.10		
	B	-0.45*	-0.05	0.03	0.43*	0.76*		

Doping in sport	T	0.43	0.88*	#	0.06	-0.32	0.65*	
	TT	0.42*	-0.28	-0.20	0.39*	0.12	-0.09	
	B	-0.09	-0.26	-0.19	-0.44*	0.13	-0.23	

Potential doping habits	T	#	#	#	#	#	#	#
	TT	0.05	0.12	0.28	0.14	0.44*	0.15	0.13
	B	0.05	0.27	0.24	0.22	-0.07	0.03	-0.35

* denotes significant correlation coefficientsat p < 0.05; # denotes relationship between variables with no variance and therefore correlation was not calculated

**Figure 1:**
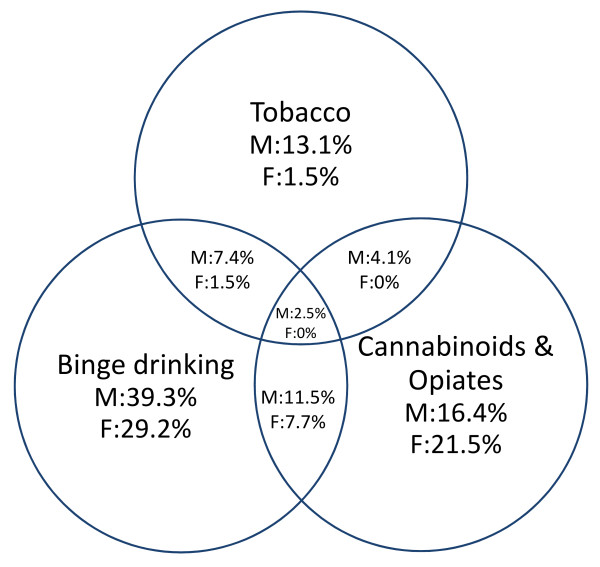
**Concomitant self-reported use of alcohol, nutritional supplements, tobacco and cannabionoids/opiates and willingness to use doping**. Percentage of reported frequency for males (M) females (F) with n = 122 and n = 65, respectively, being the 100%.

**Figure 2:**
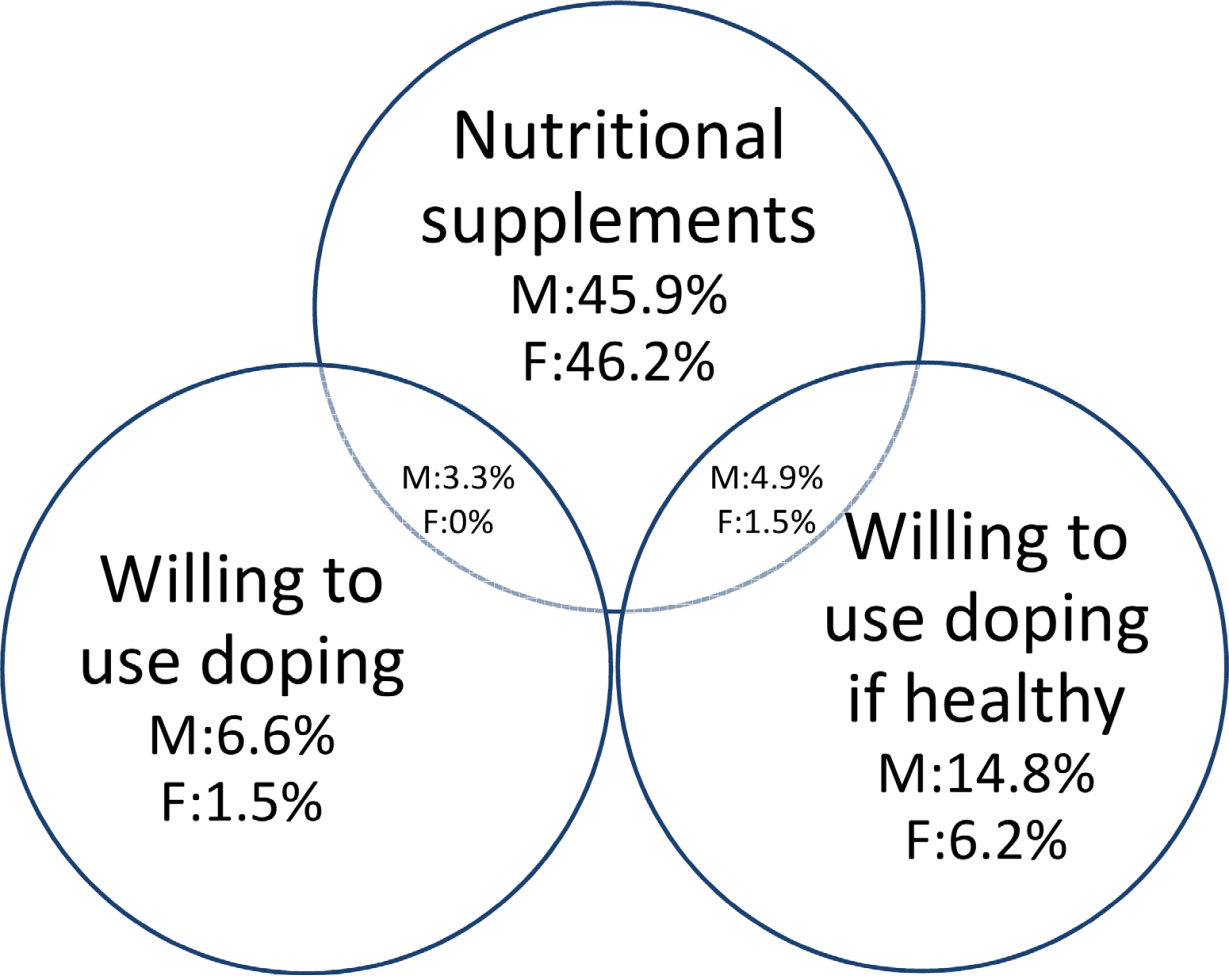
**Relationship between self-reported nutritional supplement use and willingness to use doping**. Percentage of reported frequency for males (M) females (F) with n = 122 and n = 65, respectively, being the 100%.

Figure 1 reveals an overall trend among racket sport athletes showing that the most significant overlap between self-reported use of these substances exist between binge drinking and opioid use, reaching 11% (males) and 7% (females). More precisely athletes who reported either binge drinking or opioid use were more likely to also use the other. Although these activities take place outside of the controlled sporting arena, the extent to which athletes reported these activities is concerning. Almost half of the athletes in the sample reported NS use. Interestingly, there was very little overlap between current NS use and willingness to use prohibited substances. The majority of those who indicated that they would be willing to use doping did not report current supplement use (Figure 2). However, sport- and sex-specific usage rates or expressed willingness varied greatly.

## Discussion

### Alcohol, cigarettes and drugs in Slovenian racket sports

There is evidence of alcohol abuse among male Slovenian racket sport athletes. In short, more than 20% of the studied athletes binge drink at least once a week. Binge drinking is less common in table tennis and tennis as well as among female athletes. Although Slovenian culture embraces a 'Mediterranean drinking style' in which alcohol consumption with meals is a regular daily habit, intoxication is not socially accepted in Slovenia [31]. The binging pattern we found here is probably related to both high levels of alcohol consumption in Slovenia and common alcohol consumption patterns in athletes. With a consumption rate of 14 litres of pure alcohol per capita, Slovenia is among the EU countries with the highest rate of alcohol consumption [32]. It is unsurprising that this is reflected in Slovenian athletes. Furthermore, studies have frequently found that drinking patterns in athletes exceed those of the overall population [33,34]. Both facts likely contribute to the high incidence of binging in Slovenian racket sports. We have judged the overall high rate of alcohol consumption among athletes, as the more specific explanation for the drinking behaviour of the population in question. Various theories have been proposed to explain the link between sports and alcohol use [35]. Alcohol has been associated with the rituals of relaxation and celebration in sport, which has been used to explain the higher incidence of alcohol consumption among athletes. Alcohol is also associated with risk taking, which might be more common among athletes than in the overall population [36,37]. Celebration and risk taking seem to occur most often after an athletic session, at sport-related social events, or in the company of other athletes. Post-exercise drinking may be justified by athletes with thoughts such as 'Everyone is doing it,' 'I only drink once a week,' or 'I can run/sauna it off the next morning.' In some cases, binge episodes are romanticised, and the ability to consume large quantities of alcohol is admired [38]. However, we must emphasise that such a high incidence of binge drinking is alarming and demands a serious intervention programme among Slovenian racket sport athletes.

For cigarette smoking and consumption of opiates and cannabinoids, the situation appears far better than for alcohol. Our results show a lower incidence of tobacco smoking than in the general population (23% of Slovenians smoke). The incidence of only a small percentage of the athletes of both sexes reporting that they are current smokers and reports of only occasional drug consumption suggest that this type of substance misuse is not a serious problem. Because there is evident dissimilarity between the previously discussed alcohol consumption patterns and the other two types of substance misuse studied (cigarette and drug consumption), we believe that it deserves a more detailed investigation in the future. At this point, we can only hypothesise that the athletes studied here follow the previously recognised patterns of SUM in sports worldwide. In short, substances that 'do not' affect sport performance directly (e.g., alcohol) are consumed far more often than those thought to affect performance. At the same time, athletes regularly avoid substances thought to impair and threaten achievement in sport, either by decreasing physical capacities (e.g. cigarettes) or by putting the athlete at risk of violating anti-doping codes (e.g., drug consumption) [39,40].

### Nutritional supplementation and doping-related behaviour in Slovenian racket sports

As expected from the precedence in the literature [24], more than half of the studied athletes reported NS use, and of all sports included in the study, NS are most commonly used in tennis. Although we did not study the issue more specifically, this is most likely related to the fact that tennis is the most physically challenging sport of those studied with an average energy expenditure of 7-9 kcal per minute per kilogram of body weight, which is primarily due to the greater distance covered in tennis play during bouts of activity. Additionally, a single tennis match lasts up to three hours and sometimes takes place in the heat. Of the three studied sports, tennis is the only one played on an open court [41]. Tennis tournaments range from 1 to 10 days consecutively. Because regular dietary habits rarely meet the needs of tennis players [42,43], there is a clear and relatively well-documented need for nutritional supplementation by players. Sport literature highlights the need for carbohydrate and isotonic supplementation for tennis players [44], and the athletes studied here seem to be well aware of this fact. Interestingly, of those tennis players who use nutritional supplements, more than half were advised to do so by coaches, which is a much greater percentage than the other two groups of athletes included in this study. We will discuss these findings later, together with factors related to doping.

Contrary to other studies [45], most respondents reporting current NS use do not tend towards potential doping usage. A significant proportion of athletes are of the opinion that doping is practiced in their sport with no significant difference between sports. Interestingly, this is in concordance with a 2009 WADA report [21] that noted similar findings in anti-doping testing between these sports (0.34%, 0.37%, and 0.47% of athletes with adverse analytical findings for badminton, table tennis and tennis, respectively).

As the belief that doping is practiced in their sport increases, so does the likelihood of doping behaviour in table tennis and badminton. However, as stated in the previous section, the correlation between these two variables in tennis is probably not statistically significant solely because of the smaller sample size. Evidently, athletes who more strongly think that doping is used in their sport are also more likely to use doping themselves in the future. Because similar findings have recently been published for powerlifting/weightlifting, dance-sports and synchronised swimming [28,30,46], athletes' belief that doping-behaviour is practiced in their sport should be recognised as a risk factor for doping usage. Therefore, authorities must pay special attention to this issue when tailoring anti-doping prevention and intervention programs.

Previous studies have disagreed regarding athletes' trust in their coaches on the subject of doping. Some authors have found evidence of athlete trust in their coaches' opinions [47], but studies that deal with athletes from the territory of former Yugoslavia regularly show that subjects do not rely on their coaches' opinions on this matter [28,30,46]. Therefore, although worthy of concern, the results of this present study are somewhat expected. Even more disturbing is the fact that more than 80% of the tennis and table tennis players and 60% of the badminton players report not trusting physicians' opinions on doping issues. Although this is not the first time that this problem has been noted [27], distrust of medical staff on doping issues has not been studied in detail. From our point of view and knowing the situation in this region, we believe that it can be explained as follows. Sports physicians in Slovenia are rarely professional sports doctors, but mostly are temporary involved in sports and, therefore, are only partially involved (or interested) in the athletes' training and overall development. Additionally, sports physicians are mostly focused on orthopaedic and locomotor injuries in sports and are rarely systematically educated regarding nutritional supplementation and doping [48,49]. Consequently, athletes do not consider them to be reliable, leading to low levels of trust regarding the information they provide on doping issues. These problems must not be overlooked because those who trust physicians' and coaches' opinions on doping are less prone to doping behaviour in the future [50]

### Sex differences in SUM

Female athletes are less oriented towards substance consumption than their male peers, but this is almost exclusively restricted to 'substance misuse' as previously noted. Kersey [51] reported a higher proportion of anabolic steroid usage in male athletes than in female (4.2% and 1.2%, respectively), whereas Lorente et al. [52] found that males were more prone to cannabis usage with the intention of enhancing performance. Recently, Sekulic et al. [53] studied SUM in professional ballet and showed more binge drinking in males, but a higher incidence of cigarette smoking in females. Meanwhile, recent studies have found no sex differences in nutritional supplementation [54,55]. All of these findings are consistent with this study. In short, there was no evident difference between males and females for nutritional supplementation (i.e. substance use), while females are evidently less oriented towards potential doping behaviours and alcohol consumption than their male peers (i.e. substance misuse). Although not compared statistically, it is evident that females trust their coaches' opinions regarding doping issues more than their male colleagues, which should be also noted for developing anti-doping prevention programmes in Slovenia.

### Repercussions of the anti-doping policy

Anti-doping policy focuses on preventing selected substance use in situations where such behaviourhas been deemed to result in increased athletic performance giving an unfair advantage. Drugs such as anabolic steroids that have long lasting effects and are considered 'training drugs' are prohibited both in and out of competition. Other substances, such as alcohol, marijuana and opiates, have only an in situ effect on performance and, thus, are only prohibited in competition. Furthermore, the detection-based doping policy sanctions athletes if there is evidence of a prohibited drug in their body whilst completely disregarding whether the substance found has any performance-enhancing effect on the individual. This narrow view fails to address health concerns that might arise from SUM that happens outside the regulated domain. The main pillars of the current anti-doping approach are fair play, level playing field and equal chance; only those substances that violate these principles are considered with health being secondary. The detection- and sanction-based approach to prevent doping reinforces the priority given to protecting the sport instead of protecting the athletes' health.

For anti-doping efforts to be effective there must be an attempt made to fix the weaknesses but also to reflect on the core values behind anti-doping. After all, a substance is banned if it meets any two of the three criteria: i) being performance enhancing; ii) potentially dangerous to health; and iii) against the spirit of sports as defined by the World Anti-Doping Code [56]. Thus far, the health criteria has not received the emphasis that should be warranted from the Code, which aims to ensure 'athletes' fundamental right to participate in doping-free sport and, thus, promote health, fairness and equality for athletes worldwide' (p.11) [56]; although the Athlete Biological Passport (ABP) is a notable step towards this aim. The ABP approach not only shows potential for better detection, but as it uses the same approach as in medical practice to diagnose disease based on related biomarkers, the ABP is also inherently capable of detecting compromising health patterns in addition to doping by using selected biomarkers [19].

Future anti-doping policies should address the gaps that currently exist between the testing pool and all athletes including emerging (thus not yet selected for the testing pool) athletes and those training and competing at the sub-elite level; A holistic approach to SUM that considers athletes' substance use behaviour as a whole should be used [57] in order to prevent doping and preserve not only the integrity of sports but also the athletes' health. Critical analysis of one of the three pillars of the doping ban, namely the protection of the health of athletes, points to the health risks inherently present in elite level sports along with the widespread use of acceptable substances that can also pose health risks [58]. Furthermore, that excessive alcohol or social drug use does not pose infringements upon the anti-doping rules if their use happens outside of competition is a concerning phenomenon among athletes [59-61] and gym patrons [62]. Both experts and athletes concerned agree that tailored and innovative ways are needed to deliver relevant information on performance-enhancing and illicit drugs to athletes and key stakeholders [58,63].

Trust in coaches' and physicians' opinions might be justified as Backhouse and McKenna's review [64] has shown that medical professionals, albeit against doping, possess limited knowledge of anti-doping rules and regulations. Similarly, Woods and Moynihan [49] found that less than 10% of the general practitioners (GPs) surveyed felt adequately prepared to deal with doping issues despite the fact that almost all also indicated that GPs have a role in anti-doping. The situation is even more complex for those working closely with athletes. Advising athletes on nutritional intake and medication is central to the work of athletes' support personnel. Physicians are in a particularly difficult position owing to the discrepancy between the anti-doping rules and their professional code of conduct [65].

The contextualised alcohol consumption and use of psychoactive drugs evidenced in this paper warrant further investigation. Such normalised substance use has been observed elsewhere among athletes [66]. Functional users of SUM are typically not in contact with healthcare institutions as individuals, nor are they subject to repercussions as athletes if their consumption falls outside the purview of the anti-doping regulations. However, the need for future research into contextualised, functional drug use is underscored by the fact that such a drug consumption pattern, albeit not problematic at the present, could potentially lead to unrecognised acute or chronic long-term health and psychosocial consequences. Therefore, a harm reduction approach to doping should incorporate substances beyond the Prohibited List as part of the preventive anti-doping educational effort.

### Study limitations

Limitations to generalising these results and the conclusions drawn them arise from the self-reported nature of the study and relatively small sample sizes for each sport. First, this investigation is based on subjects' self-reports. It canbe argued that subjects might not have told the truth, especially if they felt uncomfortable. However, we believe that the testing design (see Materials and Methods) decreased this possibility. Second, we must note that this study relies on a relatively small number of subjects sampled from only one country. However, because previous studies addressing SUM issues in sports noted the importance of a high proportion of respondents [24,27], we believe that the relatively small number of subjects did not significantly affect our conclusions. We studied nearly 100% of all competitive racket sports athletes in Slovenia. Finally, in some groups there is an disproportionate number of males and females (e.g., tennis), and this imbalance certainly limited statistical calculations. However, this is characteristic of female sports around the world, not only in Slovenia. Therefore, in the interpretation of these data, we have paid attention to the sex imbalance and did not interpret data only with reference to statistical findings, but also using logic. Owing to the focus in Slovenia in each discipline, most athletes compete in singles. Therefore future studies could include athletes from countries (e.g. India, Taiwan) who specialise in competing in mixed pairs to evaluate whether the observed sex differences are reduced when male and female players compete together.

## Conclusion

Findings from this study suggest that the most vulnerable subsample consists of athletes who are highly convinced that doping is present in their sport. Those with a strong belief that others in their sport are using drugs might also believe that it is a necessary practice, which in turn lead to justification for use, and are more likely to use doping themselves. At the same time, females are found to be less oriented toward substance misuse than their male peers; this is the case for all types of substances we have studied herein including cigarettes, narcotics, alcohol, regular nutritional supplement, and also - doping.

We observed low levels of athletes' trust in their coaches' and physicians' opinions on doping issues. This is an issue which should be studied in future because the underlying cause has not studied so far. Briefly, either athletes are not convinced of theirs' coaches'/medics' expertise regarding doping issues, and/or they do not believe in their good intentions. It is particularly important as previous research has shown that with increased trust in coaches and physicians, the chance that an athlete will use doping declines.

Substance misuse in sports spreads beyond those that enhance athletic performance. All of these issues should be more precisely studied in future and, if appropriately validated, incorporated into anti-doping intervention programmes. Similar to contemporary drug prevention programmes that now include performance-enhancing drugs that have spread into general population, such as anabolic steroids, pragmatic anti-doping prevention for athletes should reach beyond performance-enhancing effects and consider athletes as individuals who excel in a sport but also live a life beyond the sporting arena. Consequently, pragmatic anti-doping policies should be expanded to incorporate efforts to reduce both social and health compromising consequences associated with SUM by athletes alongside the conventional method of controlling use and the recently emerging approach of suppressing the supply. This, in turn, would help address the current anti-doping educational myopia that solely focuses on substance use in a sporting context and disregards what athletes do outside of sports. Whereas the detection-based doping testing must maintain this focus, anti-doping educational programmes have an enormous potential to see the forest from the trees, reach beyond sports and prevent doping by making a positive impact on athletes' lifestyle choices, outcome expectations and life-goals.

## Competing interests

The authors declare that they have no competing interests.

## Authors' contributions

MK designed the study, tested the subjects and drafted the manuscript. DS designed the study, performed statistical analysis and discussed data. AP contributed to the interpretation of the results and drafting the manuscript. LO developed the questionnaire drafted the manuscript and did preliminary statistical procedures. JR helped to draft the manuscript and made preliminary overview of the preceding studies. ZO participated in the study design and drafted the manuscript. All authors have read and approved the final version.
